# Self-administered transcranial direct current stimulation treatment of knee osteoarthritis alters pain-related fNIRS connectivity networks

**DOI:** 10.1117/1.NPh.10.1.015011

**Published:** 2023-03-31

**Authors:** Samuel Montero-Hernandez, Luca Pollonini, Lindsey Park, Geraldine Martorella, Hongyu Miao, Kenneth B. Mathis, Hyochol Ahn

**Affiliations:** a University of Houston, Department of Engineering Technology, Houston, Texas, United States; b University of Houston, Department of Electrical and Computer Engineering, Houston, Texas, United States; c University of Houston, Department of Biomedical Engineering, Houston, Texas, United States; d Basque Center on Cognition, Brain and Language, San Sebastian, Spain; e Florida State University, College of Nursing, Tallahassee, Florida, United States; f The University of Texas Health Science Center at Houston, McGovern Medical School, Department of Orthopedic Surgery, Houston, Texas, United States

**Keywords:** transcranial direct current stimulation, functional connectivity, pain, knee osteoarthritis, neuromodulation

## Abstract

**Significance:**

Knee osteoarthritis (OA) is a disease that causes chronic pain in the elderly population. Currently, OA is mainly treated pharmacologically with analgesics, although research has shown that neuromodulation via transcranial direct current stimulation (tDCS) may be beneficial in reducing pain in clinical settings. However, no studies have reported the effects of home-based self-administered tDCS on functional brain networks in older adults with knee OA.

**Aim:**

We used functional near-infrared spectroscopy (fNIRS) to investigate the functional connectivity effects of tDCS on underlying pain processing mechanisms at the central nervous level in older adults with knee OA.

**Approach:**

Pain-related brain connectivity networks were extracted using fNIRS at baseline and for three consecutive weeks of treatment from 120 subjects randomly assigned to two groups undergoing active tDCS and sham tDCS.

**Results:**

Our results showed that the tDCS intervention significantly modulated pain-related connectivity correlation only in the group receiving active treatment. We also found that only the active treatment group showed a significantly reduced number and strength of functional connections evoked during nociception in the prefrontal cortex, primary motor (M1), and primary somatosensory (S1) cortices. To our knowledge, this is the first study in which the effect of tDCS on pain-related connectivity networks is investigated using fNIRS.

**Conclusions:**

fNIRS-based functional connectivity can be effectively used to investigate neural circuits of pain at the cortical level in association with nonpharmacological, self-administered tDCS treatment.

## 1 Introduction

Knee osteoarthritis (OA) is a degenerative condition that destroys the cartilage inside the joint, resulting in persistent pain and functional changes, such as swelling and stiffness. It is one of the main causes of pain and physical disability in adults, affecting over 14 million people in the United States alone and millions more worldwide.^1^^,^^2^ Although knee OA can manifest at any age, it is predominant in aging adults over 50 years old, especially women.^3^

Pain caused by knee OA is mainly treated with pharmacologic agents, often producing significant adverse effects.^4^^,^^5^ Among alternative or complementary nonpharmacological approaches for pain management caused by knee OA, transcranial direct current stimulation (tDCS) has gathered interest due to its safety, noninvasiveness, and efficacy in pain reduction.^6^^–^^8^ Briefly, tDCS consists of passing a low-intensity electrical direct current between two electrodes placed on the scalp for a certain duration of time (e.g., typically 20 min) in an attempt to promote neuronal excitability within neuronal pain networks.^9^

Our research has previously shown that tDCS has a beneficial effect on clinical subjective measures of pain in knee OA patients,^10^ as well as experimental pain sensitivity.^11^^,^^12^ We reported the efficacy of home-based tDCS treatment on experimental pain sensitivity measures such as the heat pain threshold (HPTh), heat pain tolerance (HPTo), pressure pain threshold (PPT), and conditioned pain modulation (CPM), as well as its relationship with clinical pain intensity measures such as the numeric rating scale (NRS) and Western Ontario and McMaster Universities Osteoarthritis Index. Changes in NPTh, NPTo, PPT, CPM, and NRS between the active and sham groups were found to be statistically significant after the third week of treatment^12^ and 3 months from the baseline.^10^ Additionally, we found significant associations between NRS and HPTh, NRS and HPTo, and NRS and PPT. Furthermore, our group has assessed longitudinal changes in cortical hemodynamics of the tDCS treatment for pain management in a pilot study^13^ and showed the benefits of combining tDCS and mindfulness-based meditation.^14^^,^^15^ However, the mechanistic action of this neuromodulatory approach still needs to be better understood, and the use of neuroimaging offers enormous possibilities to identify the neural basis of pain perception.^16^^–^^19^

Among neuroimaging techniques, functional near-infrared spectroscopy (fNIRS) is a noninvasive optical sensing technique that measures cortical hemodynamics and that has been demonstrated to be a good fit for interrogating the brain during and after tDCS sessions.^20^ As opposed to functional magnetic resonance imaging, fNIRS bears the advantages of portability and low cost that make it particularly apt for large-sample studies carried out in nonclinical settings.

The study of pain processing with the use of fNIRS has been addressed in different works mostly focused on acute pain conditions, including but not limited to dental pain,^21^^–^^23^ induced pain in surgery,^24^^,^^25^ and acupuncture settings pain.^26^ Moreover, a growing body of literature reports nonclinical experimental studies employing thermal,^27^^–^^30^ mechanical,^28^^,^^31^ and electrical^32^^–^^36^ pain stimulation. Detailed information about approaches and experimental designs to investigate pain using fNIRS can be found in reviews published by Hu et al.^37^ and Peng et al.^38^ Although numerous fNIRS studies have explored pain-evoked brain hemodynamics, only a few of them have looked at the potential disruption of functional connectivity induced by pain. In addition, considering that pain mechanisms involve different brain areas,^17^^,^^39^ no investigations to our knowledge have analyzed the brain connectivity patterns produced by tDCS as a nonpharmacological pain treatment. For instance, Rojas et al.^26^ investigated the functional connectivity changes in 11 subjects while monitoring S1 and M1 regions during the insertion, twirl, and removal of acupuncture needles. In another work, Hu et al.^21^ explored the evolution of the resting state functional connectivity in the bilateral prefrontal cortex (PFC) and S1 in 12 patients with dentin hypersensitivity before, during, and after cold stimulation to the affected teeth. Finally, Kodama et al.^40^ analyzed the analgesic effect of the compression of myofascial trigger points on the connectivity networks recovered from the PFC with a multimodal fNIRS-EEG approach. However, none of these studies involved the use of tDCS as an alternative treatment for pain management.

According to Peyron et al.,^18^^,^^19^ neuroimaging studies focused on nociception can be divided into five categories: (i) descriptive studies aimed at the identification of patterns of brain regions involved in the processing of pain, (ii) empathy studies that explore the brain activity in the presence of unpleasant situations, (iii) clinical pain studies investigating the structural and functional brain abnormalities in clinical settings, (iv) modulation of pain studies aimed at the identification of brain regions capable of mediating the pain sensation, and (v) brain connectivity and studies focused on capturing the dynamic mechanisms of pain in terms of network analysis. Following this classification, our work can be considered to be the intersection of categories (iii), (iv), and (v).

In this study, we investigated the cross-sectional and longitudinal effects of a series of fifteen 20-min-long sessions of tDCS on functional brain connectivity in elderly individuals affected by knee OA. To our knowledge, this is the first study assessing changes in neural connections assessed using fNIRS between cohorts that underwent home-based, self-administered tDCS treatment versus sham tDCS for several weeks, and it pairs with other reports from our group focused on clinical outcomes.^10^^,^^12^^–^^15^^,^^41^^–^^43^

## 2 Materials and Methods

### 2.1 Participants

For this study, we recruited a total of 120 subjects (84 females, 66±8.3 years) suffering from knee OA. Participants were considered eligible if they had self-reported unilateral or bilateral knee OA pain, according to the American College of Rheumatology criteria.^44^ Nine subjects did not complete the study. The detailed protocol and enrollment procedures are described in our previous article reporting the primary outcomes of our study.^10^

Exclusion criteria included a history of brain surgery, brain tumor, seizure, stroke, or intracranial metal implantation; uncontrolled hypertension, heart failure, or myocardial infarction; alcohol/substance abuse; cognitive impairment; and a history of psychiatric illness. The study protocol was approved by the UTHealth Institutional Review Board prior to commencement and is registered with ClinicalTrials.gov (NCT04016272), and participants signed an informed consent form. Each participant was trained in the use of a portable tDCS device (1×1 tDCS Soterix Inc., New York, United States) to self-administer a 20-min tDCS session using 2 mA for 3 weeks (Monday to Friday) at home or a private room. The tDCS device consisted of two 5×7  cm electrodes, with the anode placed over the primary motor cortex M1 (contralateral to the affected knee) and the cathode covering the supraorbital area SO (ipsilateral to the painful knee). Participants were randomly assigned to either an active treatment or a sham group in a 1:1 ratio. For the active stimulation, the participant received a 2-mA constant direct current for 20 min according to the anode-cathode placement described before, whereas the sham group only received the electrical current stimulation for 30 s at the beginning and end of the session, with no direct current stimulation for the rest of the time. All sessions were monitored through a secure video conference platform (e.g., WebEx) to ensure that the proper brain stimulation technique was used and to monitor any adverse events.

### 2.2 Pain Experimental Protocol and fNIRS Data Collection

Functional connectivity was investigated by recording fNIRS signals during knee-localized thermal stimulation and, on a subset of participants, punctate pain stimulation. Thermal pain stimulation was applied via a commercially available thermal sensory testing machine (Medoc, Inc.) and delivered through a small thermal probe (1×1 in.) applied to the affected knee. Additionally, mechanical punctate stimulation was applied using a commercially available handheld probe (North Coast Medical, Inc.). All subjects received thermal pain stimulation, whereas only 89 received both thermal and punctate stimulation. The experimental block design consisted of applying pain stimulation for 20 s in six repetitions interleaved with resting periods of 30 s.

**Fig. 1:**
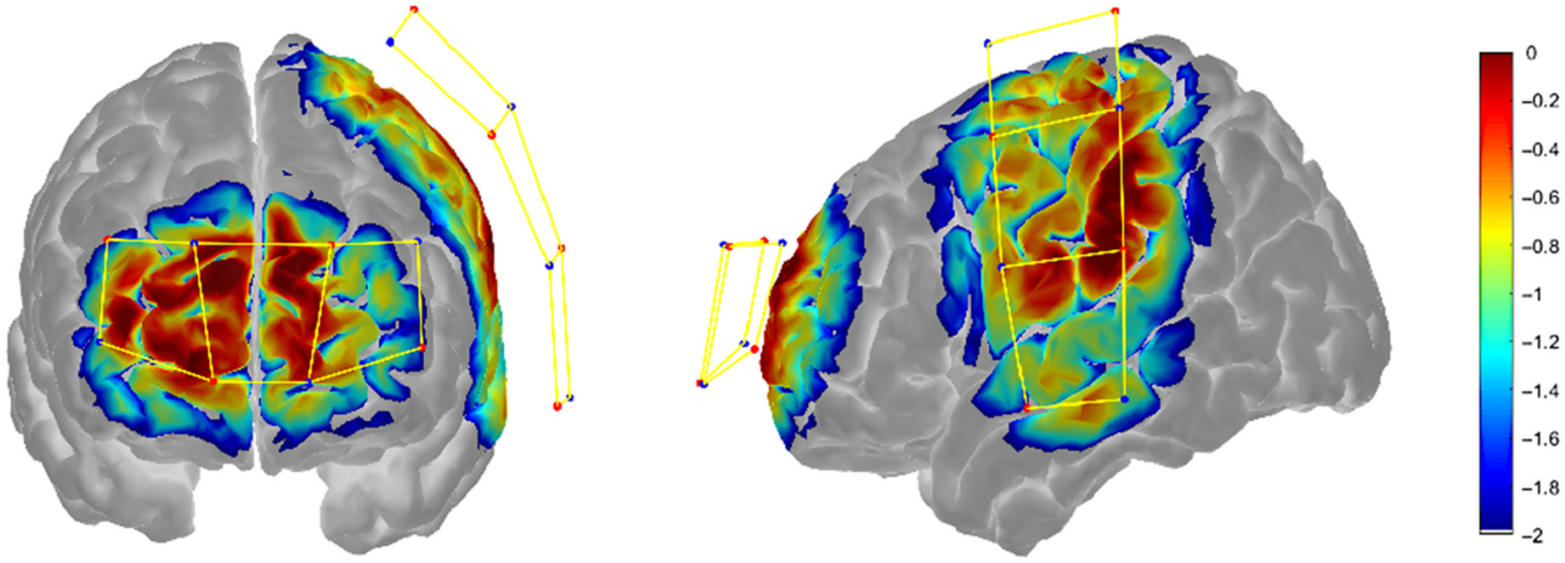
Sensitivity map of the prefrontal region and left hemisphere overlaid onto the Colin27 brain model. Sources, detectors, and channels are depicted as red circles, blue circles, and yellow bar links, respectively. The sensitivity profile was computed and displayed with AtlasViewer.

Pain-related brain responses were monitored using fNIRS at baseline (i.e., prior to any tDCS session) and after completing the first, second, and third weeks of treatment. The fNIRS device (LightNIRS, Shimadzu, Japan) consisted of eight light sources (semiconductor lasers at 780, 805, and 830 nm) and eight detectors arranged in a grid-like layout covering the PFC and primary motor (M1) and primary somatosensory (S1) contralateral to the affected knee (Fig. 1). This configuration allowed for measuring the hemodynamic activity with 20 optical channels with a source-detector distance of 30 mm approximately at 13.33 Hz.

### 2.3 Preprocessing of fNIRS Recordings

Prior to fNIRS data analysis, nine subjects were discarded because they withdrew from the study or only completed the baseline (week 0) session. Initial processing of the fNIRS data included the conversion from raw voltage to optical densities and the identification and correction of motion artifacts using the spline interpolation method.^45^ To identify recordings with poor quality data, we applied a quality control analysis on each optical channel via the QT-NIRS toolbox^46^ using the following parameters: ${\mathrm{SCI}}_{\text{threshold}}=0.8$, ${\mathrm{PSP}}_{\text{threshold}}=0.1$, and ${\mathrm{CQS}}_{\text{threshold}}=75\%$. Briefly, the scalp coupling index (SCI) and power spectrum peak (PSP) quantify the strength of the cardiac pulse detectable in a short-timed fNIRS signal (e.g., 3 to 5 s) in an optical channel, and the channel quality score (CQS) represents the time fraction of the entire recording that must have sufficient quality to consider such an optical channel acceptable. Unlike activation analyses in which each channel is associated with an activation value (e.g., a statistical p- or q-value), functional connectivity analysis is based on the statistical relationship between pairs of channels. Although pruning low-quality channels may make group-level analysis and interpretation challenging because of heterogenous connectivity matrices across subjects (i.e., networks with a different set of nodes), discarding noisy scans in their entirety improves the quality of the datasets without compromising the feasibility of subsequent analytical steps. For this reason, instead of pruning individual channels with low quality (i.e., CQS<75%), we opted to remove entire scans with fewer than five acceptable channels, as they contributed less than minimally to the functional connectivity analysis. Potential concerns arising from the inclusion of individual noisy channels are alleviated by the use of a robust correlation method^47^ (described later), which in turn is inspired by the robust regression method called autoregressive (AR) modeling using iteratively reweighted least squares (AR-IRLS).^48^ In addition, the quality metrics used in QT-NIRS have been shown to be good indicators of the performance of AR-IRLS.^49^

**Fig. 2:**
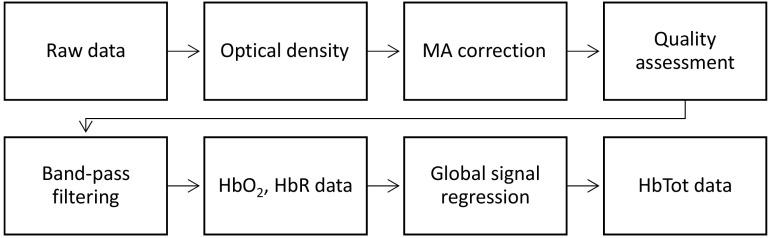
Processing pipeline of fNIRS data.

After data quality evaluation, we applied band-pass filtering (0.01 to 0.2 Hz) to remove undesired components and then converted the data to oxy-hemoglobin (${\mathrm{HbO}}_{2}$) and deoxy-hemoglobin (HbR). We then removed the remaining systemic noise by regressing out the mean response across channels for each Hb data type and scan (i.e., global system regression).^50^^–^^52^ This last step was performed due to the lack of short-separation channels in the fNIRS equipment. Finally, we computed the total hemoglobin (HbT) as the addition of the ${\mathrm{HbO}}_{2}$ and HbR. The entire processing pipeline is illustrated in Fig. 2. Data quality assessment was performed using QT-NIRS,^53^ and the rest of preprocessing, functional connectivity, and functional activation analyses were conducted using Brain AnalyzIR^54^ and Homer 2^55^ toolboxes and custom scripts in MATLAB v2021b.

### 2.4 Functional Connectivity Analysis

The brain connectivity analysis typically includes the extraction and representation of the functional connectivity networks. Extraction of the connectivity networks has been done through time-domain methods (correlation or seed-based analysis) or frequency-domain approaches (partial direct coherence or wavelet-based).^56^ The network representation approaches include graph theory methods, connectivity matrices, and atlas-based correlation maps, among others.^57^ However, both extraction and representation methods have different implications in terms of the type of retrieved information and the statistical analysis procedures. Thus, we chose the extraction and representation methods in function of the change in brain integration given the treatments (active versus sham) and the pain stimuli. From the Hb data, we computed the correlation among fNIRS channels using an AR prewhitening approach, which converts the time-series fNIRS signals to meet the statistical assumptions (independent noise terms and zero autocorrelation).^47^ Then we computed the Pearson correlation coefficients for each pair of channels across Hb types and subjects. The correlation was computed from the blocks of pain stimulation, that is, only considering each of the six 20-s trials of the stimulation periods (∼120  s). In this sense, the correlation value from channels A and B can be understood as the strength of signal association between those channels while the subject is exposed to the pain stimulation. The group-level analysis was performed over the R-coefficients using a linear mixed-effects model considering the group-week interaction for each pain stimulation type. In fNIRS connectivity, the selection of which Hb connectivity network to analyze has been debated. In this case, the similarity between the ${\mathrm{HbO}}_{2}$ and HbR networks may assist in determining whether to use either ${\mathrm{HbO}}_{2}$ or HbR (when the similarity is high) for the posterior analysis or present the results combining both Hb types. There exist specific metrics to quantify the similarity of connectivity networks; these include the graph edit distance, structural Hamming distance, and differential symmetry index (DSI).^58^ We opted to use the DSI to determine the similarity between the ${\mathrm{HbO}}_{2}$ and HbR networks because it removes the mathematical contribution of network density that increases the rate of spurious symmetry levels. Once the Hb connectivity network to be used was determined, we estimated the probability density functions (PDFs) from the distribution of the correlation values. The PDFs were computed for each stimulation type and group across weeks to quantify how different or similar the set of functional connections between the sham and active groups were. We employed a kernel density estimation with Gaussian kernels for the density estimation. The similarity between distributions densities was computed by the Hellinger distance.^59^^,^^60^ For discrete distributions, the Hellinger distance (also known as Jeffreys distance) between two distributions P and Q is defined as $H(P,Q)=\frac{1}{\sqrt{2}}{\left\|\sqrt{P}-\sqrt{Q}\right\|}_{2}$, where ${\left\|\cdot \right\|}_{2}$ denotes the Euclidean norm. We selected the Hellinger distance because it satisfies the axioms of a metric (identity, symmetry, and triangle equality) and generates values in the [0, 1] range, which simplify its interpretation.

To investigate the changes in connectivity at the regional level, we extracted the correlation values at intra- and interregional levels for PFC and primary motor and sensorimotor areas. The regional connectivity was obtained through a subnetwork analysis. First, we obtained the corresponding t values from the correlation values through the expression $t=\frac{r}{\mathrm{S.E.}}$, where $\mathrm{S.E.}=\sqrt{\frac{1-r^{2}}{n\;-2}}$ and n is the sample size. Then the statistical significance was computed and corrected using a Benjamini–Hochberg correction.

### 2.5 Statistical Analysis at the Subnetwork Level

Statistically, we analyzed functional connectivity both within and between brain regions in an attempt to gain insight into the role of subnetworks involved in functional organization patterns in pain management. Specifically, we described the regional connectivity using mean correlation and mean node degree of subnetworks. The mean correlation represents the strength of connectivity, whereas the mean node degree represents the total wiring cost of a subnetwork.^61^ In functional connectivity networks, the division into subnetworks can be done according to different criteria, for instance, anatomical divisions, channels density coverage, and well-known brain circuitry, among others.^62^^,^^63^ Here we choose a trade-off between the regions of interest relevant to this study and the channel density coverage of our fNIRS setup, thus exploring connectivity in two regions: the PFC and the lateral region that includes the primary motor cortex (M1) and primary sensorimotor (S1) area. We applied the statistical analysis on both measures according to the following scheme. First, the Shapiro–Wilk test was used to examine the normality assumption of data distribution, and then two sample t-tests or Wilcoxon Rank sum tests (when normality was not met) were used to compare the active group with the sham group.

## 3 Results

### 3.1 Data Quality

**Fig. 3:**
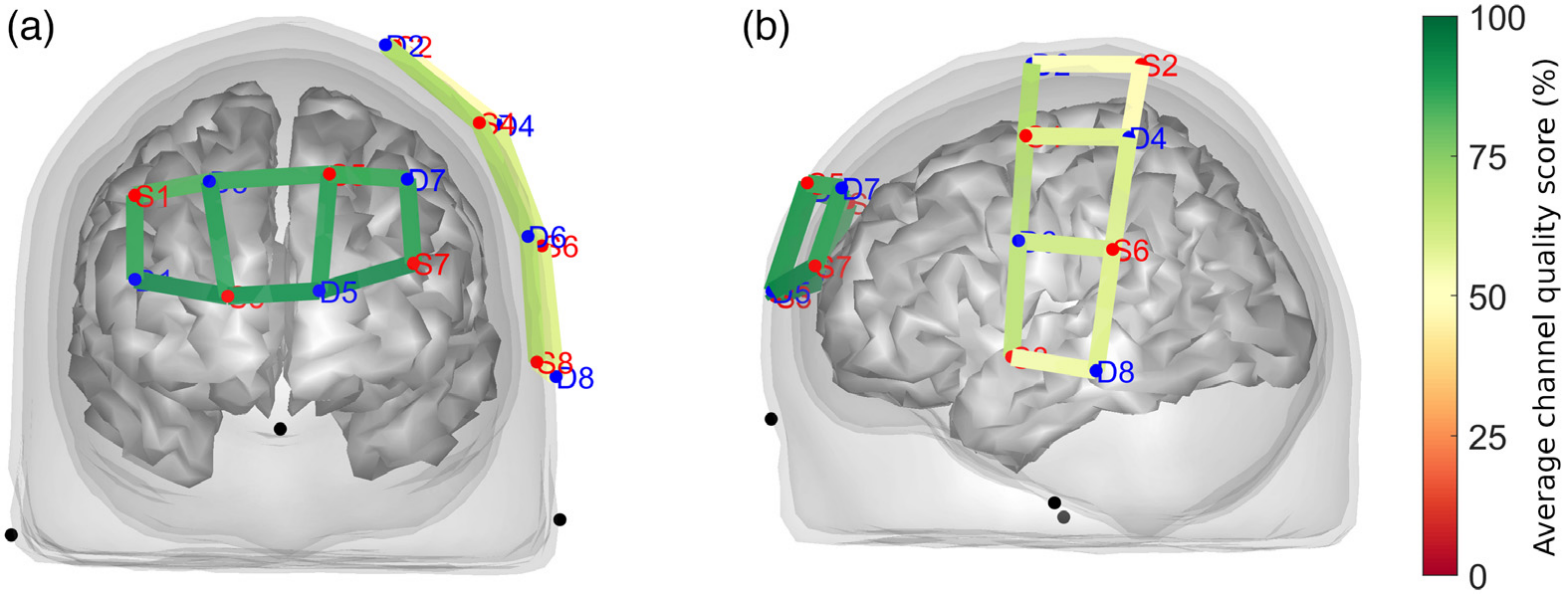
Resulting data quality assessment using QT-NIRS after triaging excessively noisy scans. Optical channels covering (a) the prefrontal region exhibited higher quality (CQS close to 95%) than channels over (b) the sensory-motor brain area (CQS close to 60%).

Our approach to data quality evaluation (i.e., triaging scans with less than five acceptable channels) resulted in the exclusion of 56 out of 799 recordings (or 7% of the total) from further processing. The resulting CQS averaged across the screened dataset is shown in Fig. 3. Expectedly, optical channels over the PFC had a visibly higher quality (∼95%) compared with those probing the primary motor and somatosensory cortical areas (∼60%), where hair obtrusion contributed to an overall lower signal quality.

### 3.2 Time Series

**Fig. 4:**
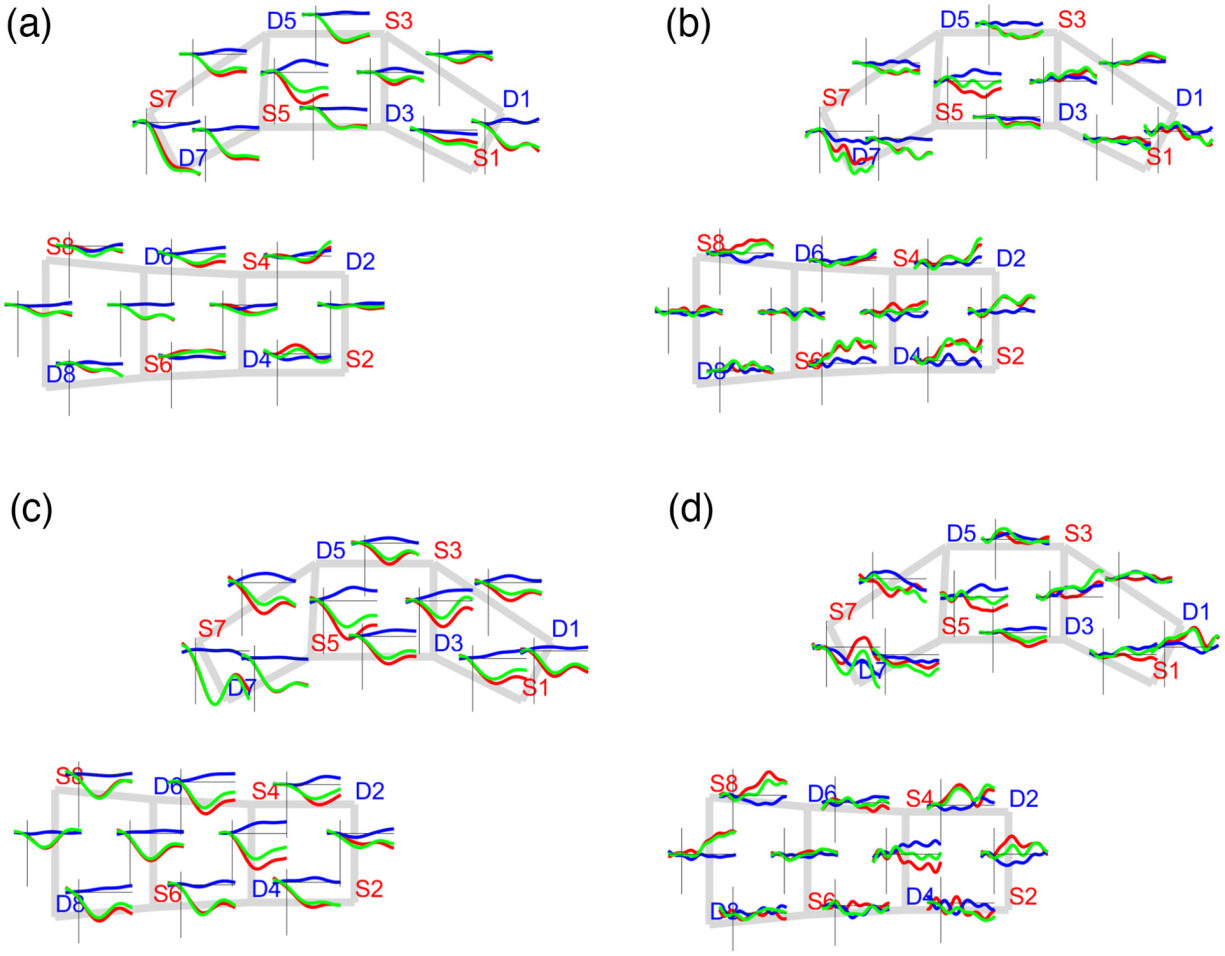
Global block-averaged response for sham (a) before and (b) after and active (c) before and (d) after applying the global signal regression to remove the global physiological effects of pain punctate stimulation (punctate). ${\mathrm{HbO}}_{2}$, HbR, and HbT signals are shown in red, blue, and green, respectively.

In addition to the quality assessment, the removal of the global systemic signals unveils the hemodynamic changes while discarding the effects of physiology from the scalp when subjects are exposed to pain stimulation. Figure 4 shows the block-averaged ${\mathrm{HbO}}_{2}$, HbR, and HbT group-level responses.

After removing the global systemic components, hemodynamic changes were expectedly observed in the transition from resting to stimulation periods. Increases in HbT were observed on channels covering the motor and somatosensory regions, whereas negative HbT changes took place on channels covering the PFC. In other words, the signals from channels covering the PFC exhibited an anticorrelated temporal profile in contrast to those from the primary motor and somatosensory regions.

**Fig. 5:**
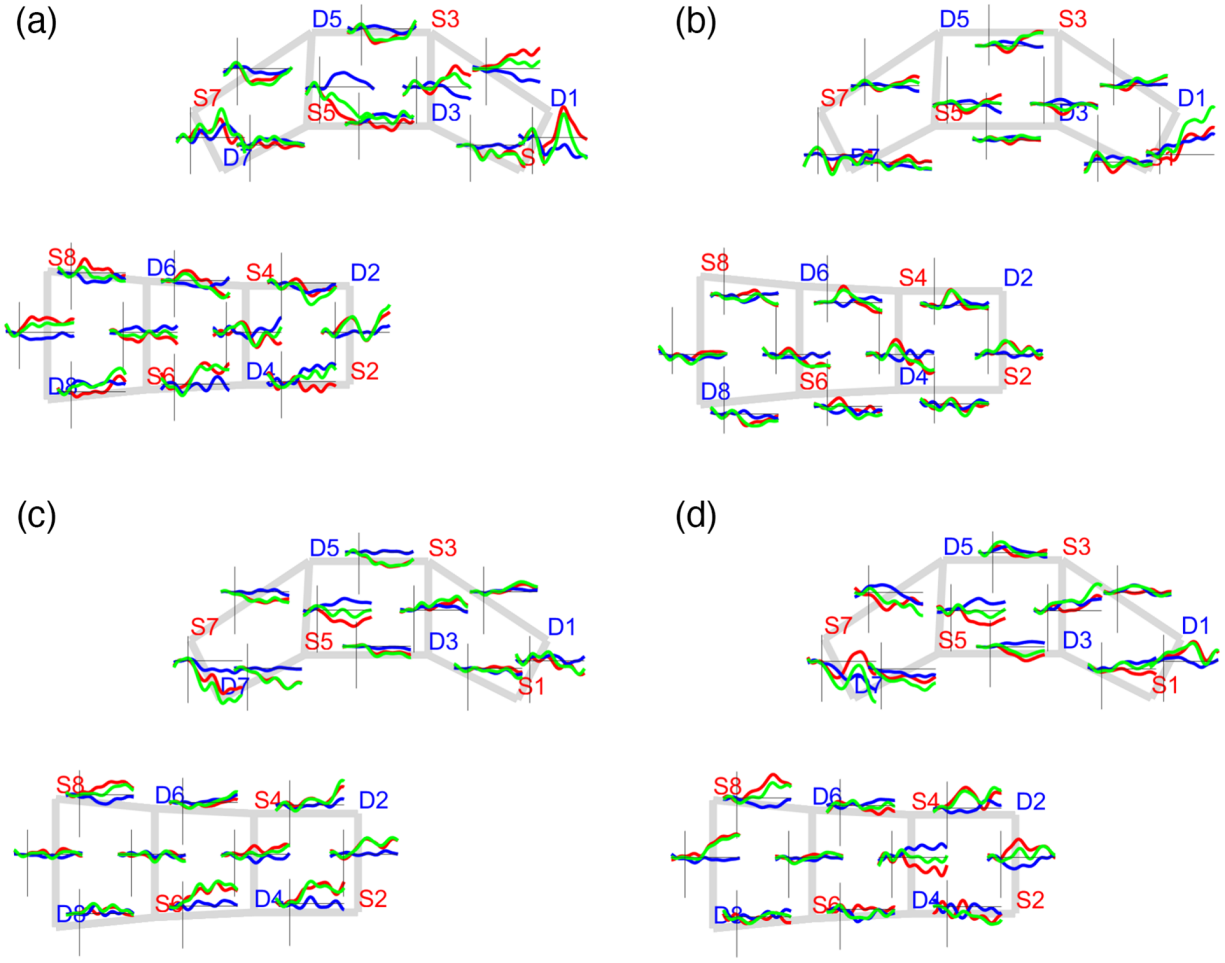
Block-averaged group-level response. The average hemodynamic response of pain stimulation during baseline (week 1). Thermal stimuli: (a) sham and (b) active; punctate stimuli (c) sham and (d) active. ${\mathrm{HbO}}_{2}$, HbR, and HbT signals are shown in red, blue, and green, respectively.

Figure 5 shows the block-averaged hemodynamic temporal profile at the group level for the thermal and punctate pain stimuli at the beginning of the treatment. Different cases of activation and negative activation patterns arose from the pain-evoked responses on both thermal and punctate stimuli. Activation responses are defined as an ${\mathrm{HbO}}_{2}$ increase concomitant to an HbR decrease pattern, and negative activation responses show the opposite behavior, that is, an ${\mathrm{HbO}}_{2}$ decrease simultaneous with an HbR increase.

With regards to the thermal stimulation type, a considerable set of channels on the primary motor and somatosensory regions showed activation patterns (sham group: S8-D8, S8-D6, S4-D6, S6-D4, and S2-D2; active group: S4-D6, S4-D2, S4-D4, S2-D2, and S6-D4), whereas channels over the PFC exhibited a negative activation response (sham group: S5-D5, S5-D3, S3-D5, and S1-D3; active group: S5-D5, S3-D3, and S1-D3).

With respect to the punctate stimulation, the sham group exhibited more activated channels covering the primary motor and somatosensory regions than the active group (sham: S8-D6, S4-D4, S2-D2, S6-D4, and S2-D4; active: S8-D6, S4-D2, S8-D8, and S2-D2). A similar number of negative activation cases were found on both the sham (S7-D5, S5-D5, and S5-S7) and active (S5-D7, S5-D5, and S5-D3) groups over the PFC region. In contrast, we observed a set of channels with opposite responses to the previous ones, that is, with an activation profile on the PFC and a negative activation response on the primary motor and sensorimotor regions. However, the number of those cases was small, and it was also investigated during the functional connectivity analysis. In general, the activation versus negative activation cases suggested an anticorrelated relationship between PFC and the primary motor and sensorimotor areas that was quantified by connectivity analysis.

### 3.3 Functional Connectivity

Functional connectivity among channels was quantified by computing a correlation between the ${\mathrm{HbO}}_{2}$, HbR, and HbT time series considering the blocks of stimulation periods. At the scan level, we obtained 743 connectivity networks, and at the group level we computed a set of 48 connectivity networks (#stimuli x #groups x #weeks x #Hb types; 2×2×4×3). The DSI metric was calculated considering only their significant connections (q-value<0.05). Then we based our analysis only on the HbT results due to the low ${\mathrm{HbO}}_{2}-\mathrm{HbR}$ symmetry value (mean = 0.02 and std = 0.01). Figure S3 in the Supplementary Material shows the DSI results for all connectivity networks at the scan level, and Figs. S1 and S2 in the Supplementary Material present the group-level HbT connectivity maps (across stimuli, groups, and weeks) and contrasts maps (week 2 to week 0), respectively.

Because we focused our analysis on the variation of functional connectivity strength, we present the functional connectivity models as connectivity matrices. In a connectivity matrix, rows and columns represent the fNIRS channels and the elements $c_{i,j}$ represent the R coefficient value between the channels i and j. Note that this representation does not disentangle the directionality of the relationship, but only its strength. Because we did not focus on the directionality of the existing relationships, all of the resulting connectivity matrices are symmetric.

### 3.4 Thermal Stimulation

**Fig. 6:**
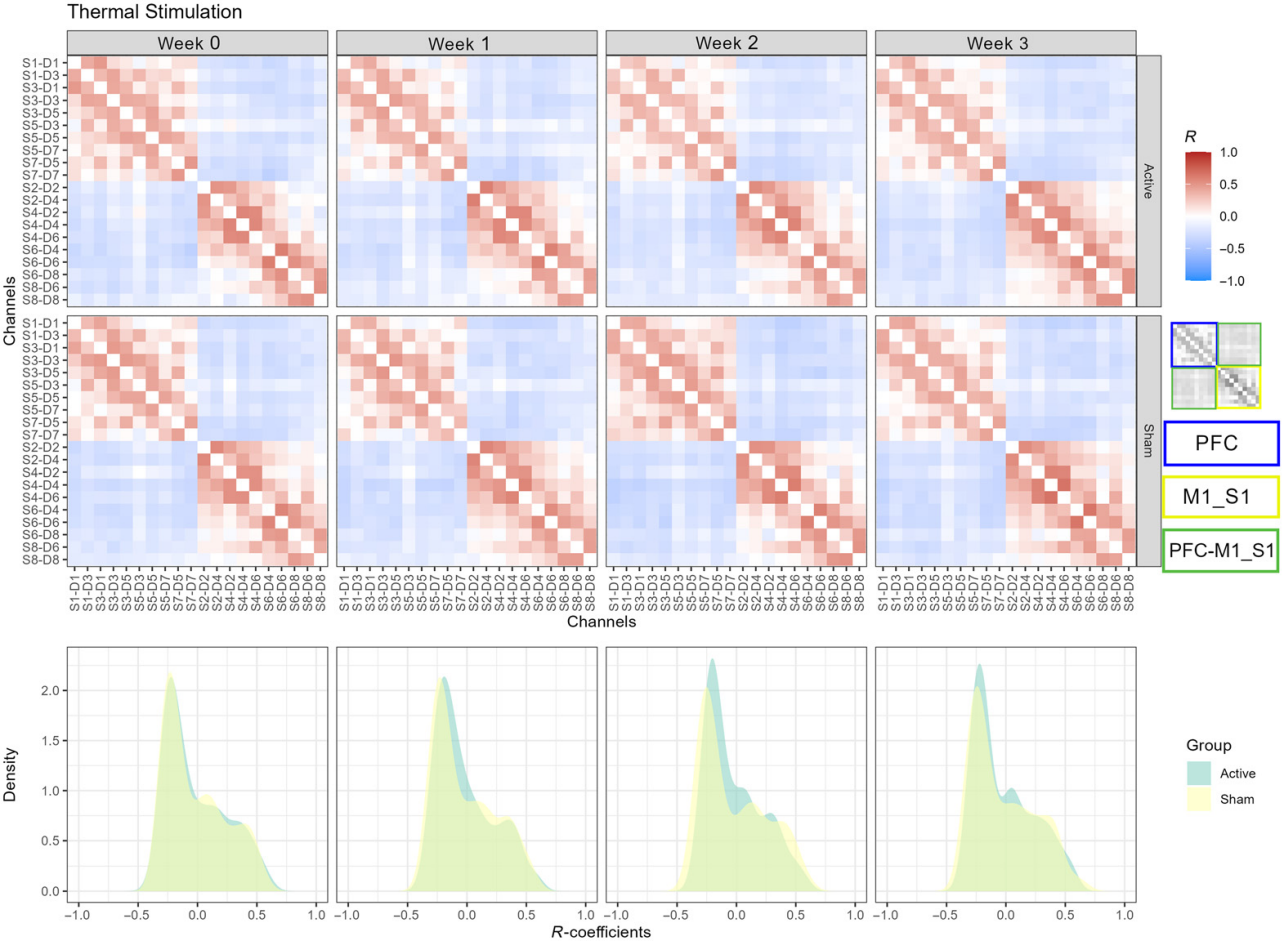
Group-level connectivity models of thermal stimulation. Color-coded correlation values for active (first row) and sham (second row) groups across sessions (columns 1 to 4). PFC, M1_S1, and PFC-M1_S1 regions are indicated by the blue, yellow, and green rectangles. The estimated probability densities of the active and sham groups are shown across sessions (bottom row).

In Fig. 6, we present the HbT connectivity matrices derived from group-level analysis. For each matrix, we show the connectivity matrix and the estimated probability densities. We observed a high-positive intraconnectivity in the PFC and M1_S1 regions in every session for both active and sham groups, as depicted in the first and second rows in Fig. 6 and Fig. S1 in the Supplementary Material. Notably, we found a negative between-regions connectivity. The positive correlation among neighboring channels denotes a localized hemodynamic response, suggesting a modularity organization of the investigated brain regions, whereas the anticorrelated connections exhibit the cooperation between the PFC and primary motor and sensorimotor regions. In addition to the regional organization analysis, we computed the similarity of the connectivity models between groups based on the distributions of the R-values computed from each model. Table 1 lists the H-distances describing the longitudinal changes across weeks. The minimum distance between the densities was observed at week 0 with H=0.0250, whereas the maximum separation was observed in week 2 (H=0.1254) (Fig. 6, bottom row). The low H-distance found at baseline denotes that the intra- and interregional connectivity in both groups exhibits high similar connectivity strength in response to the thermal stimulation at the beginning of the treatment. On the other hand, the maximum H-distance was found in week 2, suggesting the time point with the maximum effect of tDCS treatment. Additionally, important changes can be observed in the density shapes. For the sham group during week 2, the shape shows a more bimodal profile due to a decrease in the density around correlation values near zero, which indicates that some of the weak connections improved their strength. In terms of the network organization, it may be caused by the increasing modularity pattern in the M1_S1 area from week 0 to week 2 [Fig. 6 (second row, M1_S1 area, week 0 versus week 2); Fig. S1 (first row, column 3) in the Supplementary Material]. Additionally, we observed an increase in the interregional PFC-M1_S1 negative connections from week 0 to week 2 [Fig. S1 (first row, columns 1 and 3) in the Supplementary Material]. This increment is confirmed by the statistical contrast map from week 2 to week 0 shown in Fig. S2 (first row, column 1) in the Supplementary Material. On the contrary, the opposite effects were observed for the active group. The increase in the density around zero correlation values suggests that some of the functional links weaken their correlation. This reduced effect in connectivity could be mostly observed in the intraregional connectivity of PFC [Fig. 6 (first row, columns 1 and 3); Fig. S1 (second row, columns 1 and 3) in the Supplementary Material]. The decrease in PFC connectivity was also captured by the contrast map shown in Fig. S2 (first row, column 2, PFC area) in the Supplementary Material.

**Table 1:** Hellinger distances between sham and active correlation distributions for the HbT connectivity networks. H(A,B)=0 expresses identical distributions. Maximum differences between distributions across weeks are indicated in bold.

	Thermal	Punctate
Week 0	0.0250	0.0366
Week 1	0.0678	0.0833
Week 2	**0.1254**	**0.1215**
Week 3	0.0661	0.0627

To investigate the interregional connectivity, we applied a threshold of |R|≥0.3 (where |·| is the absolute value) to the statistically significant functional links (q-value<0.05). We selected the value of 0.3 because it effectively removes the weak connections in the intraregional connectivity while keeping the interregion links. Figure S4 in the Supplementary Material illustrates the R-values of the connections along with the statistical significance before and after applying the |R|≥0.3 threshold. Then we obtained the mean correlation and node degree within and between the PFC and M1_S1 regions. Table 2 summarizes the statistical comparison between the sham and active groups for every connectivity model at the subnetwork level. At the beginning of the treatment, no statistically significant differences were found. We found significant differences between the sham and active groups in both week 1 and week 2. Only the interregion connectivity network (PFC-M1_S1) exhibited differences between groups during week 1 in terms of correlation. On the other hand, significant differences were found in the PFC intra- and interconnectivity (PFC-M1_S1) subnetworks for both correlation and degree metrics. No significant differences between the active and sham groups in correlation or degree metrics were found in the last week of the treatment.

**Table 2:** Comparison between groups across weeks for thermal stimulation. Correlation and degree values computed from subnetwork analysis. Statistically significant (*p* < 0.05) correlation and degree values are indicated in bold.

Session	Subnetwork	Active	Sham	p-value
Week 0	M1_S1 degree	21.87 (6.66)	22.06 (9.05)	0.86
	M1_S1 correlation	0.15 (0.14)	0.13 (0.19)	0.87
	PFC degree	20.00 (10.00)	21.50 (9.50)	0.58
	PFC correlation	0.12 (0.17)	0.10 (0.14)	0.42
	PFC–M1_S1 degree	39.00 (21.50)	46.00 (23.50)	0.29
	PFC–M1_S1 correlation	−0.15 (0.14)	−0.16 (0.15)	0.91
Week 1	M1_S1 degree	19.50 (12.00)	21.00 (10.00)	0.44
	M1_S1 correlation	0.13 (0.17)	0.16 (0.20)	0.46
	PFC degree	20.00 (9.00)	21.00 (10.00)	0.22
	PFC correlation	0.11 (0.09)	0.12 (0.15)	0.54
	PFC–M1_S1 degree	39.00 (18.00)	39.00 (22.00)	0.22
	**PFC–M1_S1 correlation**	**−0.13 (0.10)**	**−0.15 (0.11)**	**0.04**
Week 2	M1_S1 degree	20.00 (8.00)	19.50 (12.00)	0.92
	M1_S1 correlation	0.15 (0.09)	0.15 (0.15)	0.91
	**PFC degree**	**17.00 (8.00)**	**20.00 (10.00)**	**<0.01**
	**PFC correlation**	**0.09 (0.11)**	**0.15 (0.17)**	**<0.01**
	**PFC–M1_S1 degree**	**37.00 (18.00)**	**40.00 (24.00)**	**0.01**
	**PFC–M1_S1 correlation**	**−0.13 (0.10)**	**−0.16 (0.14)**	**0.01**
Week 3	M1_S1 degree	21.61 (7.92)	22.68 (8.12)	0.33
	M1_S1 correlation	0.16 (0.13)	0.16 (0.18)	0.81
	PFC degree	21.00 (7.00)	20.00 (9.00)	0.23
	PFC correlation	0.11 (0.14)	0.11 (0.12)	0.44
	PFC–M1_S1 degree	40.00 (17.00)	43.00 (23.00)	0.15
	PFC–M1_S1 correlation	−0.14 (0.09)	−0.17 (0.15)	0.26

### 3.5 Mechanical Punctate Stimulation

**Fig. 7:**
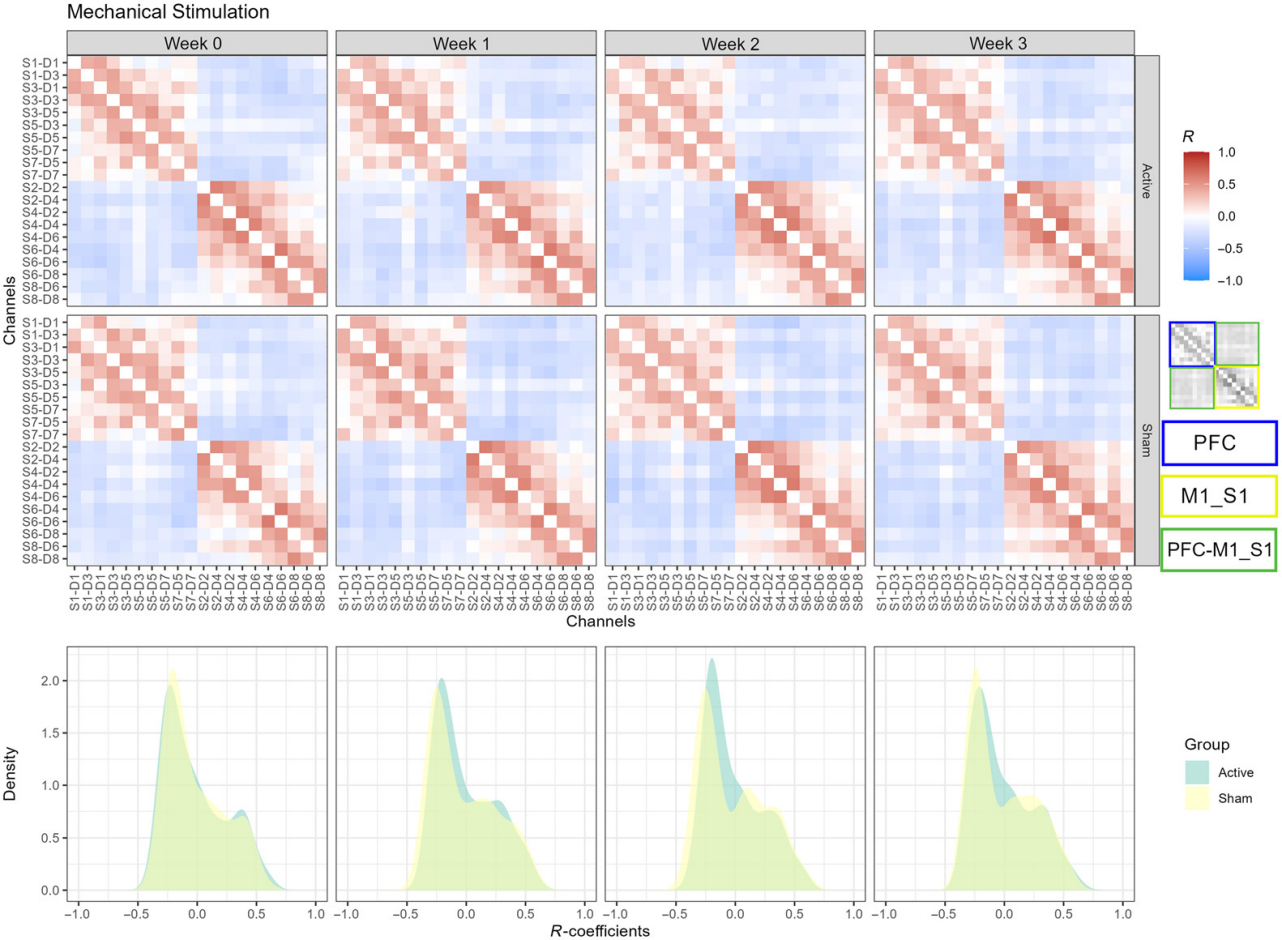
Group-level connectivity models of mechanical punctate stimulation. Color-coded correlation values for active (first row) and sham (second row) groups across sessions (columns 1 to 4). PFC, M1_S1, and PFC-M1_S1 regions are indicated by the blue, yellow, and green rectangles. The estimated probability densities of the active and sham groups are shown across sessions (bottom row).

As in the thermal stimulation, a clear positive and negative statistical association was observed in the intra- and interregional brain connectivity, respectively. In general, the M1 and S1 areas exhibited more organized correlated responses for both the sham and active groups than the associations in the PFC (Fig. 7, first and second rows). Additionally, a decrease in the positive correlations was observed in the PFC area for the active group between week 0 and week 2 (Fig. 7, first row, columns 1 and 3), confirmed by their statistical contrast (week 2 to week 0) shown in Fig. S2 (last row columns 1 and 3) and Fig. S2 (second row column 2) in the Supplementary Material. Moreover, an increment in the negative interregional PFC-M1_S1 connections was observed in the sham group between weeks 0 and 2 (Fig. 7 second row, columns 1 and 3) and was also captured by their statistical contrast [Fig. S1 (third row, columns 1 and 3); Fig. S2 (second row, column 1) in the Supplementary Material]. In terms of the density of the correlations, a high overlap is observed between the sham and active groups at the baseline (Fig. 7, last row, first column) with a Hellinger distance of 0.0366. For week 1, the separation between groups increased for the positive and negative correlations; however, in week 2, the distance seemed to be larger for the negative associations but not for the positive correlations (Fig. 7, last row, second and third columns). Finally, the distance between densities started to decrease between groups during week 3. The evolution of the correlations from the sham group showed that, in week 2, the peaks for positive correlations became more evident, suggesting a decrease in weak positive connections (close to zero correlations). Also from week 2, the density of positive correlations for both the sham and active groups started to overlap again, as at the beginning of the treatment. This suggests that, for the punctate pain stimulation, one important difference between groups was captured by the interregional brain connectivity, which exhibited mostly negative correlations.

Table 3 presents the comparison between groups across weeks for the punctate pain stimulation at the subnetwork level (correlation and degree values). We applied the same thresholding procedure (|R|≥0.3) as in the subnetwork analysis. No statistical differences were found at the baseline between the sham and active groups for any subnetwork. However, we found statistically significant differences between the sham and active groups during week 1 for PFC intraregion and PFC-M1_S1 interregion connectivity in the correlation and degree domains.

**Table 3:** Comparison between groups across weeks for thermal stimulation. Correlation and degree values computed from subnetwork analysis. Statistically significant (*p* < 0.05) correlation and degree values are indicated in bold.

	Subnetwork	Active	Sham	p-value
Week 0	M1_S1 degree	22.21 (9.23)	20.78 (9.94)	0.36
	M1_S1 correlation	0.18 (0.21)	0.12 (0.17)	0.21
	PFC degree	23.00 (8.00)	21.00 (11.00)	0.40
	PFC correlation	0.10 (0.12)	0.09 (0.11)	0.87
	PFC–M1_S1 degree	41.00 (30.00)	36.00 (32.00)	0.40
	PFC–M1_S1 correlation	−0.12 (0.17)	−0.12 (0.16)	0.26
Week 1	M1_S1 degree	24.00 (11.00)	21.50 (11.50)	0.07
	M1_S1 correlation	0.17 (0.20)	0.14 (0.18)	0.25
	**PFC degree**	**20.00 (7.00)**	**22.00 (5.00)**	**<0.01**
	**PFC correlation**	**0.09 (0.13)**	**0.16 (0.15)**	**0.03**
	**PFC–M1_S1 degree**	**37.50 (25.00)**	**45.00 (23.00)**	**<0.01**
	**PFC–M1_S1 correlation**	**−0.13 (0.12)**	**−0.17 (0.17)**	**0.01**
Week 2	M1_S1 degree	23.00 (13.00)	21.00 (16.00)	0.62
	M1_S1 correlation	0.17 (0.17)	0.14 (0.19)	0.46
	PFC degree	19.00 (7.00)	20.00 (11.00)	0.13
	PFC correlation	0.09 (0.09)	0.10 (0.11)	0.10
	PFC–M1_S1 degree	39.00 (22.00)	38.00 (31.00)	0.23
	PFC–M1_S1 correlation	−0.14 (0.13)	−0.15 (0.20)	0.16
Week 3	M1_S1 degree	22.00 (8.00)	21.50 (11.00)	0.60
	M1_S1 correlation	0.18 (0.14)	0.17 (0.14)	0.84
	PFC degree	19.50 (7.50)	18.50 (11.00)	0.38
	PFC correlation	0.10 (0.11)	0.10 (0.10)	0.80
	PFC–M1_S1 degree	40.00 (18.50)	44.00 (23.00)	0.26
	PFC–M1_S1 correlation	−0.14 (0.10)	−0.17 (0.15)	0.13

## 4 Discussion

In this study, we investigated the offline (poststimulation) effects of tDCS therapy on brain connectivity patterns associated with pain stimulation. Using fNIRS, our study monitored the brain hemodynamic responses following a self-administered 3-week tDCS therapy in older adults suffering from knee OA. Previously, our group investigated the use of alternative (nonpharmacological) treatments for pain management in chronic knee pain cohorts.^11^^,^^13^^–^^15^^,^^41^^–^^43^ Our results showed changes in the connectivity at inter- and intraregional levels between the tDCS active and sham groups across weeks based on two metrics (i.e., correlation and average node degree), tracing the changes in the circuitry associated with punctate and thermal pain stimulation.

Functional responses during pain stimulation have been explored using different neuroimaging modalities.^19^ Moreover, the use of tDCS has been previously investigated in pain management^64^^–^^66^ and in combination with fNIRS.^20^ The tDCS offline effects can last, in some cases, several days after the end of a tDCS session,^67^^,^^68^ so here we report the induced changes by tDCS on functional connectivity in response to pain management during a longitudinal tDCS treatment of 15 sessions over 3 weeks and compare them against a sham tDCS therapy.

Throughout the entire experimental activity, we observed average hemodynamic responses (without the global systemic components) in agreement with the literature that associated the sympathetic skin response observed in pain-induced studies.^34^ Also, we found concomitant decrease–increase responses in ${\mathrm{HbO}}_{2}-\mathrm{HbR}$ on channels covering PFC, whereas the opposite increase–decrease ${\mathrm{HbO}}_{2}-\mathrm{HbR}$ changes were found in M1 and S1 regions. These hemodynamic patterns have been previously reported in studies for detecting brain responses associated with thermal,^21^^,^^23^^,^^30^^,^^69^ mechanical,^70^ and electrical^34^^,^^35^ pain stimulation. Hemodynamic responses to pain stimulation have been observed in cortical and subcortical regions, such as insular, primary, and secondary somatosensory (S1 and S2) cortices, anterior cingulate cortex (ACC), supplementary motor area cortices, and PFC (Bas 10, 45-47) region.^18^ Although the functional connectivity of the mechanisms of pain may include associated processes (because pain experience can trigger concomitant processes, such as cognitive, anticipatory, emotional, and cognitive, among others), the maximum similarity value (measured by the Hellinger distance) was found between the tDCS active and sham groups at the baseline (week 0) for both stimulation types (see Table 1), whereas the minimum similarity between the active and sham connectivity networks was found during week 2. Considering the active group, weeks 1 and 3 exhibited similar Hellinger distances to the baseline values. In terms of correlation and average node degree, no statistically significant differences were found in week 0 between the active and sham groups for thermal or punctate stimulation types. With regards to thermal stimulation, we found a significant difference between the tDCS active and sham groups in weeks 1 and 2 (see Table 2), with the maximum effect of the tDCS therapy in week 2. Specifically, we found statistically significant differences in PFC-M1_S1 on week 1 and PFC and PFC-M1_S1 networks on week 2. In the case of the punctate stimulation, PFC and PFC-M1_S1 networks exhibited significantly different patterns between groups in week 1 with a major effect on the number of functional connection than the connectivity strength, which has been observed in patients with hip and knee OA.^71^^,^^72^

We observed greater PFC average node degree and correlation values for the tDCS sham group. The PFC network from the sham group exhibited not only more functional connections but also a higher correlation among its nodes. The PFC, specifically BA 10, has been associated with an accelerated deoxygenated activity observed during nociceptive processing.^32^^,^^34^^,^^73^ Also, it has been shown that PFC plays a role in the inhibited connectivity exhibited by the default mode network during cognitive processing,^74^ which can be caused due to the role of BA 10 in attentional processes, such as switching focus or paying attention to pain.^75^ Contrary to the sham tDCS, the active tDCS therapy reflected weak connectivity in PFC when dealing with pain management, which can be an indication of a weaker attentional process to pain.^76^^,^^77^

For the M1 and S1 regions, we observed a decrease in the (negative) connectivity strength and the number of connections for the active tDCS group. The stronger (positive) correlation and the greater number of links in the sham tDCS group can be translated into a more interacting interregion connectivity between the PFC and the M1 and S1 regions for dealing with the pain experience. Several positive and negative coactivations between BA 10 and other brain regions, such as S1, S2, ACC, the insular cortex, dorsolateral PFC, ventrolateral PFC, premotor area, and subcortical structures such as the thalamus and striatum, have been reported in the literature.^38^ Also the existence of different networks associated with stronger connectivity values in response to acupuncture stimulation has been shown.^26^ Additionally, the existence of a negative correlation among different brain regions, including M1, S1, and PFC, has been observed in clinical dental pain settings using fNIRS^21^ and in OA patients with MRI,^78^ which supports the negative associations that we found in the interregional network connectivity.

Our results should be interpreted considering the following limitations. First, the tDCS treatment was applied in a home-based self-administered fashion, which may cause variation in the levels of efficacy across subjects. To reduce such potential variations, the participants were properly trained in the use of the tDCS device at the baseline and remotely supervised at each stimulation session by trained research staff to ensure the correct technique. Second, short separation channels were not included in the fNIRS probe due to inherent technical features of the fNIRS device used in this study (i.e., optodes could not be placed at a distance of <30  mm). However, we included a mean global systemic regression step to minimize the presence of systemic noise. Third, after the data quality assessment, we remove a subset of recordings instead of removing channels. Unlike functional studies in which every channel is associated with an activation metric, connectivity analysis always requires pairs of channels to estimate their association. Hence, we avoided pruning individual channels and opted to remove noisy scans in their entirety to obtain homogenous connectivity matrices across subjects. Considering that the large sample size (n=120) yielded a total of 799 recordings, the exclusion of a small subset of poor-quality recordings (56 scans or 7% of total) did not compromise the objective of this study. Finally, the fNIRS cap was carefully fitted utilizing anatomical landmarks (such as the nasion, inion, and preauricular points) as spatial references to maximize the repeatability of the optode placement. We also utilized a 3D digitizer (Polhemus Patriot) to acquire the spatial coordinates of all optodes and anatomical landmarks in 115 subjects. However, the digitization task could only be trusted for 97 subjects due to intermittent electromagnetic noise affecting the digitizer readings in our experimental facility. Notwithstanding this issue, trustworthy digital placements showed that the placement could be repeated within a few millimeters (median = 12.2 mm, IQR = 9.5 to 15.2 mm), as illustrated in Fig. S5 in the Supplementary Material.

Despite these limitations, we believe that our findings provide avenues for future research studies on pain-related brain function. Our results suggest that the use of a tDCS therapy produces large-scale neuronal changes that can be captured by a functional connectivity analysis based on fNIRS. Also active and sham tDCS treatments were associated with distinguishable hemodynamic responses to pain stimulation. Finally, tDCS-derived connectivity changes could be related to the efficacy of the therapy in modulating the neural pain circuitry during nociception in knee OA patients.

## 5 Conclusion

We have investigated the effect of tDCS therapy in a pain-evoked experience at the brain connectivity level using fNIRS. We have found different longitudinal brain connectivity patterns between active tDCS therapy for pain management and sham tDCS in 120 participants across three weeks of therapy. Our results confirmed a decrease in connectivity in PFC and S1 and M1 regions after the first and second weeks of therapy for punctate and thermal pain stimulation, which contrasted with an increase in connectivity relations and strength shown by the sham tDCS group. The contrast may be associated with the tDCS therapy that promotes the modulation of PFC, S1, and M1 in the pain experience of participants. These results open the door for new investigations in the context of pain management, such as the refinement of tDCS parameters for individualized treatment.

## Code, Data, and Materials Availability

The MATLAB scripts and functions used to process the dataset and analyze the results reported in this paper utilized the functionality of the QT-NIRS, Homer2, and NIRS Brain AnalyzIR toolboxes and can be found in Refs. 53, 79, and 80. The datasets generated and/or analyzed during the current study are available from the corresponding author upon reasonable request.

## References

[1] Wallace I. J. et al., “Knee osteoarthritis has doubled in prevalence since the mid-20^th^ century,” Proc. Natl. Acad. Sci. U. S. A. 114(35), 9332–9336 (2017). 10.1073/pnas.1703856114 28808025 PMC5584421

[2] Deshpande B. R. et al., “Number of persons with symptomatic knee osteoarthritis in the US: impact of race and ethnicity, age, sex, and obesity,” Arthritis Care Res. (Hoboken) 68(12), 1743–1750 (2016). 10.1002/acr.22897 27014966 PMC5319385

[3] Cristina de Oliveira N. et al., “Lifestyle and pain in women with knee osteoarthritis,” Am. J. Lifestyle Med. 13(6), 606–610 (2017). 10.1177/1559827617722112 31662727 PMC6796231

[4] Welsch P. et al., “Opioids for chronic osteoarthritis pain: an updated systematic review and meta-analysis of efficacy, tolerability and safety in randomized placebo-controlled studies of at least 4 weeks double-blind duration,” Eur. J. Pain 24(4), 685–703 (2020). 10.1002/ejp.1522 31876347

[5] Krebs E. E. et al., “Effect of opioid vs nonopioid medications on pain-related function in patients with chronic back pain or hip or knee osteoarthritis pain: the SPACE randomized clinical trial,” JAMA 319(9), 872–882 (2018). JAMAAP 0098-7484 10.1001/jama.2018.0899 29509867 PMC5885909

[6] Bikson M. et al., “Safety of transcranial direct current stimulation: evidence based update 2016,” Brain Stimul. 9(5), 641–661 (2016). 10.1016/j.brs.2016.06.004 27372845 PMC5007190

[r7] Mori F. et al., “Effects of anodal transcranial direct current stimulation on chronic neuropathic pain in patients with multiple sclerosis,” J. Pain 11(5), 436–442 (2010). 10.1016/j.jpain.2009.08.011 20018567

[8] Simis M. et al., “Investigation of central nervous system dysfunction in chronic pelvic pain using magnetic resonance spectroscopy and noninvasive brain stimulation,” Pain Pract. 15(5), 423–432 (2015). 10.1111/papr.12202 24799153 PMC4216781

[9] Pacheco-Barrios K. et al., “Methods and strategies of tDCS for the treatment of pain: current status and future directions,” Expert Rev. Med. Devices 17(9), 879–898 (2020).1743-4440 10.1080/17434440.2020.1816168 32845195 PMC7674241

[10] Martorella G. et al., “Self-administered transcranial direct current stimulation for pain in older adults with knee osteoarthritis: a randomized controlled study,” Brain Stimul. 15(4), 902–909 (2022). 10.1016/j.brs.2022.06.003 35690388 PMC9387776

[11] Ahn H. et al., “Bayesian analysis of the effect of transcranial direct current stimulation on experimental pain sensitivity in older adults with knee osteoarthritis: randomized sham-controlled pilot clinical study,” J. Pain Res. 11, 2071–2082 (2018). 10.2147/JPR.S173080 30310309 PMC6166765

[12] Martorella G. et al., “Efficacy of home-based transcranial direct current stimulation on experimental pain sensitivity in older adults with knee osteoarthritis: a randomized, sham-controlled clinical trial,” J. Clin. Med. 11(17), 5209 (2022). 10.3390/jcm11175209 36079139 PMC9457351

[r13] Pollonini L. Miao H. Ahn H., “Longitudinal effect of transcranial direct current stimulation on knee osteoarthritis patients measured by functional infrared spectroscopy: a pilot study,” Neurophotonics 7(2), 025004 (2020). 10.1117/1.NPh.7.2.025004 32411812 PMC7203445

[r14] Pollonini L. et al., “Functional near-infrared spectroscopy to assess central pain responses in a nonpharmacologic treatment trial of osteoarthritis,” J. Neuroimaging 30(6), 808–814 (2020). JNERET 1051-2284 10.1111/jon.12782 32896933 PMC7719610

[15] Ahn H. et al., “Efficacy of combining home-based transcranial direct current stimulation with mindfulness-based meditation for pain in older adults with knee osteoarthritis: a randomized controlled pilot study,” J. Clin. Neurosci. 70, 140–145 (2019). 10.1016/j.jocn.2019.08.047 31421990

[16] Lindsay N. M. et al., “Brain circuits for pain and its treatment,” Sci. Transl. Med. 13(619), eabj7360 (2021). STMCBQ 1946-6234 10.1126/scitranslmed.abj7360 34757810 PMC8675872

[r17] Iannetti G. D. Mouraux A., “From the neuromatrix to the pain matrix (and back),” Exp. Brain Res. 205(1), 1–12 (2010). EXBRAP 0014-4819 10.1007/s00221-010-2340-1 20607220

[r18] Peyron R. Laurent B. García-Larrea L., “Functional imaging of brain responses to pain. A review and meta-analysis (2000),” Neurophysiol. Clin. 30(5), 263–288 (2000). NCLIE4 0987-7053 10.1016/S0987-7053(00)00227-6 11126640

[19] Peyron R. Fauchon C., “Functional imaging of pain,” Revue Neurol. 175(1–2), 38–45 (2019). RENEAM 0035-3787 10.1016/j.neurol.2018.08.006 30318262

[20] Patel R. et al., “Systematic review of combined functional near-infrared spectroscopy and transcranial direct-current stimulation studies,” Neurophotonics 7(2), 020901 (2020). 10.1117/1.NPh.7.2.020901 32607389 PMC7315225

[21] Hu X. et al., “Brain functional changes before, during, and after clinical pain,” J. Dent. Res. 97(5), 523–529 (2018). JDREAF 0022-0345 10.1177/0022034517750136 29324076 PMC9096192

[r22] Hu X. S. et al., “Feasibility of a real-time clinical augmented reality and artificial intelligence framework for pain detection and localization from the brain,” J. Med. Internet Res. 21(6), e13594 (2019). 10.2196/13594 31254336 PMC6625219

[23] Racek A. J. et al., “Different brain responses to pain and its expectation in the dentalchair,” J. Dent. Res. 94(7), 998 (2015). JDREAF 0022-0345 10.1177/0022034515581642 25904140 PMC9096194

[24] Gélinas C. et al., “Toward a new approach for the detection of pain in adult patients undergoing cardiac surgery: near-infrared spectroscopy—a pilot study,” Hear. Lung J. Acute Crit. Care 39(6), 485–493 (2010). 10.1016/j.hrtlng.2009.10.018 20561850

[25] Kussman B. D. et al., “Capturing pain in the cortex during general anesthesia: near infrared spectroscopy measures in patients undergoing catheter ablation of arrhythmias,” PloS One 11(7), e0158975 (2016). POLNCL 1932-6203 10.1371/journal.pone.0158975 27415436 PMC4944937

[26] Rojas R. F. et al., “Cortical network response to acupuncture and the effect of the Hegu point: an FNIRS study,” Sensors 19(2), 394 (2019). 10.3390/s19020394 30669377 PMC6359459

[27] Barati Z. Zakeri I. Pourrezaei K., “Functional near-infrared spectroscopy study on tonic pain activation by cold pressor test,” Neurophotonics 4(1), 015004 (2017). 10.1117/1.NPh.4.1.015004 28386576 PMC5358549

[r28] Becerra L. et al., “Diffuse optical tomography of pain and tactile stimulation: activation in cortical sensory and emotional systems,” Neuroimage 41(2), 252–259 (2008). NEIMEF 1053-8119 10.1016/j.neuroimage.2008.01.047 18394924 PMC2728450

[r29] Hong K. S. et al., “Classification of somatosensory cortex activities using fNIRS,” Behav. Brain Res. 333, 225–234 (2017). BBREDI 0166-4328 10.1016/j.bbr.2017.06.034 28668280

[30] Fernandez Rojas R. Huang X. Ou K. L., “A machine learning approach for the identification of a biomarker of human pain using fNIRS,” Sci. Rep. 9, 5645 (2019). SRCEC3 2045-2322 10.1038/s41598-019-42098-w 30948760 PMC6449551

[31] Bartocci M. et al., “Pain activates cortical areas in the preterm newborn brain,” Pain 122(1–2), 109–117 (2006). PAINDB 0304-3959 10.1016/j.pain.2006.01.015 16530965

[32] Peng K. et al., “Morphine attenuates fNIRS signal associated with painful stimuli in the medial frontopolar cortex (medial BA 10),” Front. Hum. Neurosci. 12, 394 (2018). 10.3389/fnhum.2018.00394 30349466 PMC6186992

[r33] Re R. et al., “Cerebral cortex activation mapping upon electrical muscle stimulation by 32-channel time-domain functional near-infrared spectroscopy,” Adv. Exp. Med. Biol. 789, 441–447 (2013). AEMBAP 0065-2598 10.1007/978-1-4614-7411-1_59 23852527

[r34] Yücel M. A. et al., “Specificity of hemodynamic brain responses to painful stimuli: a functional near-infrared spectroscopy study,” Sci. Rep. 5, 9469 (2015). SRCEC3 2045-2322 10.1038/srep09469 25820289 PMC4377554

[r35] Peng K. et al., “Using prerecorded hemodynamic response functions in detecting prefrontal pain response: a functional near-infrared spectroscopy study,” Neurophotonics 5(1), 011018 (2017). 10.1117/1.NPh.5.1.011018 29057285 PMC5641587

[36] Aasted C. M. et al., “Frontal lobe hemodynamic responses to painful stimulation: a potential brain marker of nociception,” PloS One 11(11), e0165226 (2016). POLNCL 1932-6203 10.1371/journal.pone.0165226 27806119 PMC5091745

[37] Hu X. S. Nascimento T. D. DaSilva A. F., “Shedding light on pain for the clinic: a comprehensive review of using functional near-infrared spectroscopy to monitor its process in the brain,” Pain 162(12), 2805–2820 (2021). PAINDB 0304-3959 10.1097/j.pain.0000000000002293 33990114 PMC8490487

[38] Peng K. et al., “Brodmann area 10: collating, integrating and high level processing of nociception and pain,” Prog. Neurobiol. 161, 1–22 (2018). PGNBA5 0301-0082 10.1016/j.pneurobio.2017.11.004 29199137 PMC5826795

[39] Hall M. et al., “Pain induced changes in brain oxyhemoglobin: a systematic review and meta-analysis of functional NIRS studies,” Pain Med. 22(6), 1399–1410 (2021). 10.1093/pm/pnaa453 33659994

[40] Kodama K. et al., “Analgesic effects of compression at trigger points are associated with reduction of frontal polar cortical activity as well as functional connectivity between the frontal polar area and insula in patients with chronic low back pain: a randomized trial,” Front. Syst. Neurosci. 13, 68 (2020). 10.3389/fnsys.2019.00068 PMC6863771 31798422

[41] Ahn H. et al., “Feasibility and efficacy of remotely supervised cranial electrical stimulation for pain in older adults with knee osteoarthritis: a randomized controlled pilot study,” J. Clin. Neurosci. 77, 128–133 (2020). 10.1016/j.jocn.2020.05.003 32402609 PMC7308202

[r42] Ahn H. et al., “Efficacy of transcranial direct current stimulation over primary motor cortex (anode) and contralateral supraorbital area (cathode) on clinical pain severity and mobility performance in persons with knee osteoarthritis: an experimenter- and participant-blinded, randomized, sham-controlled pilot clinical study,” Brain Stimul. 10(5), 902–909 (2017). 10.1016/j.brs.2017.05.007 28566193 PMC5568498

[43] Ahn H. et al., “Home-based self-administered transcranial direct current stimulation in older adults with knee osteoarthritis pain: an open-label study,” J. Clin. Neurosci. 66, 61–65 (2019). 10.1016/j.jocn.2019.05.023 31153751

[44] Altman R. et al., “Development of criteria for the classification and reporting of osteoarthritis: classification of osteoarthritis of the knee,” Arthritis Rheum. 29(8), 1039–1049 (1986). 10.1002/art.1780290816 3741515

[45] Scholkmann F. et al., “How to detect and reduce movement artifacts in near-infrared imaging using moving standard deviation and spline interpolation,” Physiol. Meas. 31(5), 649 (2010). PMEAE3 0967-3334 10.1088/0967-3334/31/5/004 20308772

[46] Montero Hernandez S. Pollonini L., “NIRSplot: a tool for quality assessment of fNIRS scans,” in Biophotonics Congr.: Biomed. Opt. 2020, p. BM2C.5, The Optical Society (2020).

[47] Santosa H. et al., “Characterization and correction of the false-discovery rates in resting state connectivity using functional near-infrared spectroscopy,” J. Biomed. Opt. 22(5), 055002 (2017). JBOPFO 1083-3668 10.1117/1.JBO.22.5.055002 28492852 PMC5424771

[48] Barker J. W. Aarabi A. Huppert T. J., “Autoregressive model based algorithm for correcting motion and serially correlated errors in fNIRS,” Biomed. Opt. Express 4(8), 1366 (2013). BOEICL 2156-7085 10.1364/BOE.4.001366 24009999 PMC3756568

[49] Santosa H. et al., “Quantitative comparison of correction techniques for removing systemic physiological signal in functional near-infrared spectroscopy studies,” Neurophotonics 7(3), 035009 (2020). 10.1117/1.NPh.7.3.035009 32995361 PMC7511246

[50] Liu T. T. Nalci A. Falahpour M., “The global signal in fMRI: nuisance or information?,” Neuroimage 150, 213–229 (2017). NEIMEF 1053-8119 10.1016/j.neuroimage.2017.02.036 28213118 PMC5406229

[r51] Lanka P. Bortfeld H. Huppert T. J., “Correction of global physiology in resting-state functional near-infrared spectroscopy,” Neurophotonics 9(3), 035003 (2022). 10.1117/1.NPh.9.3.035003 35990173 PMC9386281

[52] Zhang Y. et al., “Eigenvector-based spatial filtering for reduction of physiological interference in diffuse optical imaging,” J. Biomed. Opt. 10(1), 011014 (2005). JBOPFO 1083-3668 10.1117/1.1852552 15847580

[53] https://github.com/lpollonini/qt-nirs.

[54] Santosa H. et al., “The NIRS brain AnalyzIR toolbox,” Algorithms 11(5), 73 (2018). 10.3390/a11050073 1748-7188 PMC11218834 38957522

[55] Huppert T. J. et al., “HomER: a review of time-series analysis methods for near-infrared spectroscopy of the brain,” Appl. Opt. 48(10), D280–D298 (2009). APOPAI 0003-6935 10.1364/AO.48.00D280 19340120 PMC2761652

[56] Bastos A. M. Schoffelen J. M., “A tutorial review of functional connectivity analysis methods and their interpretational pitfalls,” Front. Syst. Neurosci. 9, 175 (2016). 10.3389/fnsys.2015.00175 26778976 PMC4705224

[57] Sporns O., Networks of the Brain, MIT Press, Cambridge, Massachusetts (2011).

[58] Montero-Hernandez S. et al., “Estimating functional connectivity symmetry between oxy- and deoxy-haemoglobin: implications for fNIRS connectivity analysis,” Algorithms 11(5), 70 (2018).1748-7188 10.3390/a11050070

[59] Jeffreys H., “An invariant form for the prior probability in estimation problems,” Proc. R. Soc. Lond. A. Math. Phys. Sci. 186(1007), 453–461 (1946). 10.1098/rspa.1946.0056 20998741

[60] Chung J. K. et al., “Measures of distance between probability distributions,” J. Math. Anal. Appl. 138(1), 280–292 (1989). JMANAK 0022-247X 10.1016/0022-247X(89)90335-1

[61] Rubinov M. Sporns O., “Complex network measures of brain connectivity: uses and interpretations,” Neuroimage 52(3), 1059–1069 (2010). NEIMEF 1053-8119 10.1016/j.neuroimage.2009.10.003 19819337

[62] Marrelec G. Fransson P., “Assessing the influence of different ROI selection strategies on functional connectivity analyses of fMRI data acquired during steady-state conditions,” PloS One 6(4), e14788 (2011). POLNCL 1932-6203 10.1371/journal.pone.0014788 21533283 PMC3076321

[63] Sohn W. S. et al., “Influence of ROI selection on resting functional connectivity: an individualized approach for resting fMRI analysis,” Front. Neurosci. 9, 280 (2015).1662-453X 10.3389/fnins.2015.00280 26321904 PMC4531302

[64] Fregni F. et al., “A sham-controlled, phase II trial of transcranial direct current stimulation for the treatment of central pain in traumatic spinal cord injury,” Pain 122(1–2), 197–209 (2006). PAINDB 0304-3959 10.1016/j.pain.2006.02.023 16564618

[r65] Fenton B. W. et al., “A preliminary study of transcranial direct current stimulation for the treatment of refractory chronic pelvic pain,” Brain Stimul. 2(2), 103–107 (2009). 10.1016/j.brs.2008.09.009 20633407

[66] Fagerlund A. J. Hansen O. A. Aslaksen P. M., “Transcranial direct current stimulation as a treatment for patients with fibromyalgia: a randomized controlled trial,” Pain 156(1), 62–71 (2015). PAINDB 0304-3959 10.1016/j.pain.0000000000000006 25599302

[67] Jones K. T. Gözenman F. Berryhill M. E., “The strategy and motivational influences on the beneficial effect of neurostimulation: a tDCS and fNIRS study,” Neuroimage 105, 238–247 (2015). NEIMEF 1053-8119 10.1016/j.neuroimage.2014.11.012 25462798 PMC4262534

[68] Besson P. et al., “Concurrent anodal transcranial direct-current stimulation and motor task to influence sensorimotor cortex activation,” Brain Res. 1710, 181–187 (2019). BRREAP 0006-8993 10.1016/j.brainres.2019.01.003 30610875

[69] Becerra L. et al., “Diffuse optical tomography activation in the somatosensory cortex: specific activation by painful vs. non-painful thermal stimuli,” PloS One 4(11), e8016 (2009). POLNCL 1932-6203 10.1371/journal.pone.0008016 19956637 PMC2778627

[70] Holper L. et al., “Physiological effects of mechanical pain stimulation at the lower back measured by functional near-infrared spectroscopy and capnography,” J. Integr. Neurosci. 13(1), 121–142 (2014). 10.1142/S0219635214500071 24738542

[71] Baliki M. N. et al., “A preliminary fMRI study of analgesic treatment in chronic back pain and knee osteoarthritis,” Mol. Pain 4, 47 (2008). 10.1186/1744-8069-4-47 18950528 PMC2584040

[72] Parks E. L. et al., “Brain activity for chronic knee osteoarthritis: dissociating evoked pain from spontaneous pain,” Eur. J. Pain 15(8), 843.e1–843.e14 (2011). 10.1016/J.EJPAIN.2010.12.007 PMC3113642 21315627

[73] Becerra L. et al., “Brain measures of nociception using near-infrared spectroscopy in patients undergoing routine screening colonoscopy,” Pain 157(4), 840–848 (2016). PAINDB 0304-3959 10.1097/j.pain.0000000000000446 26645550 PMC4794375

[74] Fox M. D. et al., “The human brain is intrinsically organized into dynamic, anticorrelated functional networks,” Proc. Natl. Acad. Sci. U. S. A. 102(27), 9673–9678 (2005). 10.1073/pnas.0504136102 15976020 PMC1157105

[75] Burgess P. W. Dumontheil I. Gilbert S. J., “The gateway hypothesis of rostral prefrontal cortex (area 10) function,” Trends Cogn. Sci. 11(7), 290–298 (2007). TCSCFK 1364-6613 10.1016/j.tics.2007.05.004 17548231

[76] Peyron R. et al., “Haemodynamic brain responses to acute pain in humans: sensory and attentional networks,” Brain 122(9), 1765–1780 (1999). BRAIAK 0006-8950 10.1093/brain/122.9.1765 10468515

[77] Baliki M. N. et al., “Beyond feeling: chronic pain hurts the brain, disrupting the default-mode network dynamics,” J. Neurosci. 28(6), 1398–1403 (2008). JNRSDS 0270-6474 10.1523/JNEUROSCI.4123-07.2008 18256259 PMC6671589

[78] Baliki M. N. et al., “Brain morphological signatures for chronic pain,” PloS One 6(10), e26010 (2011). POLNCL 1932-6203 10.1371/journal.pone.0026010 22022493 PMC3192794

[79] http://openfnirs.org/software/homer/.

[80] https://github.com/huppertt/nirs-toolbox.

