# Supplementary material for: Self-administered transcranial direct current stimulation treatment of knee osteoarthritis alters pain-related fNIRS connectivity networks
Source: Neurophotonics. 2023 Mar 31;10(1):015011. doi: 10.1117/1.NPh.10.1.015011 (PMC10063907; doi:10.1117/1.NPh.10.1.015011)
Supplement: Supplementary file 1 [file NPh_010_015011_SD001.pdf]

Self-administered tDCS Treatment of Knee Osteoarthritis Alters Pain-related fNIRS Connectivity Networks

Samuel Montero-Hernandez[a], Luca Pollonini[a,b,c], Lindsey Park[d], Geraldine Martorella[d], Hongyu Miao[d], Kenneth B. Mathis[e] and Hyochol Ahn[d]

[a] Department of Engineering Technology, University of Houston, 4800 MLK Blvd., Houston, TX 77204 USA
[b] Department of Electrical and Computer Engineering, University of Houston, 4800 MLK Blvd., Houston, TX 77204 USA
[c] Department of Biomedical Engineering, University of Houston, 4800 MLK Blvd., Houston, TX 77204 USA
[d] College of Nursing, Florida State University, 98 Varsity Way, Tallahassee, FL 32306 USA
[e] Department of Orthopedic Surgery McGovern Medical School at The University of Texas Health Science Center at Houston 6431 Fannin Houston, TX 77030

Supplemental Figures

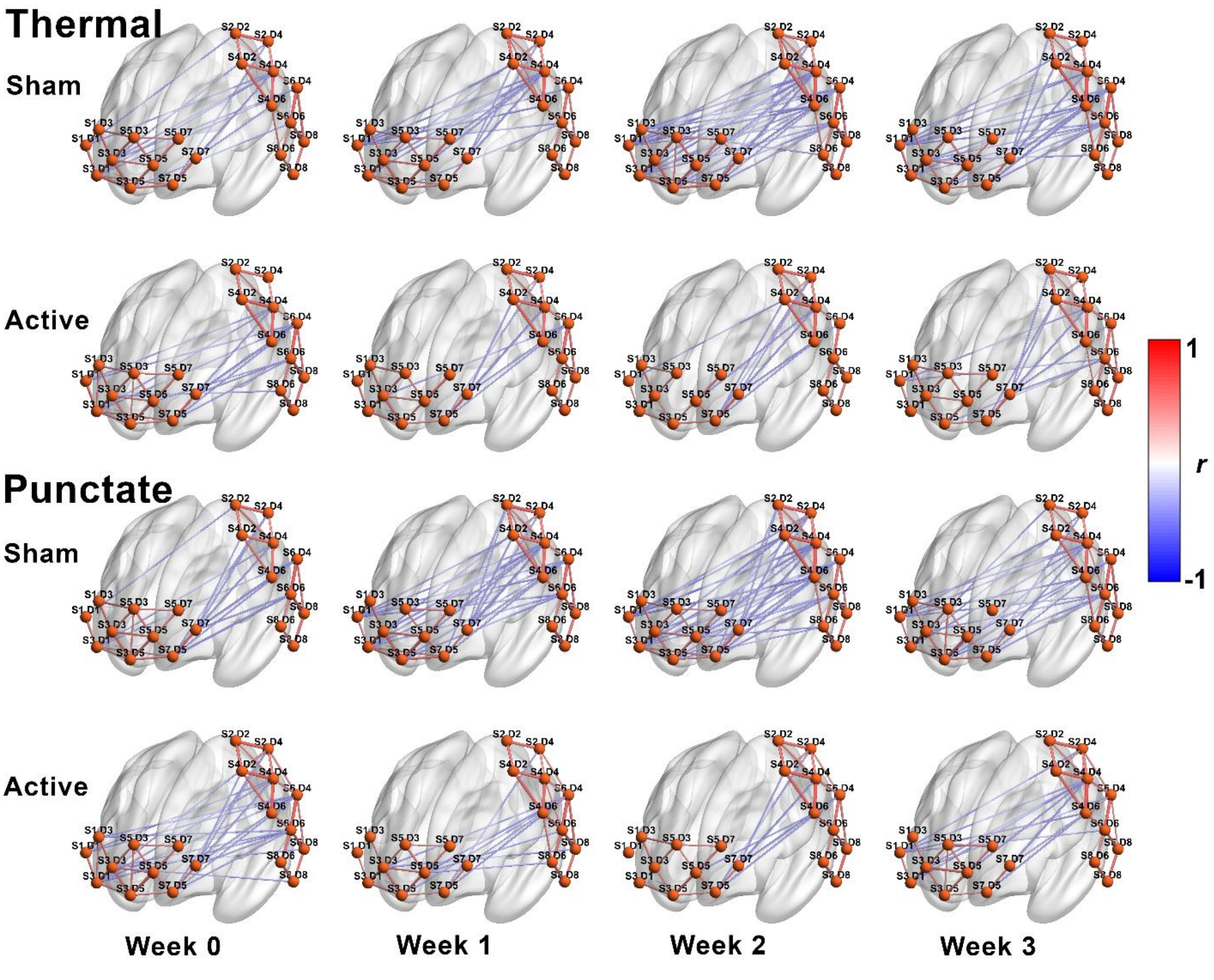

Figure S1 Group-level connectivity maps across weeks (columns) for thermal-sham, thermal-active, punctate-sham, and punctate-active groups (rows). Positive and negative correlation values are encoded in the color and size of the network links.

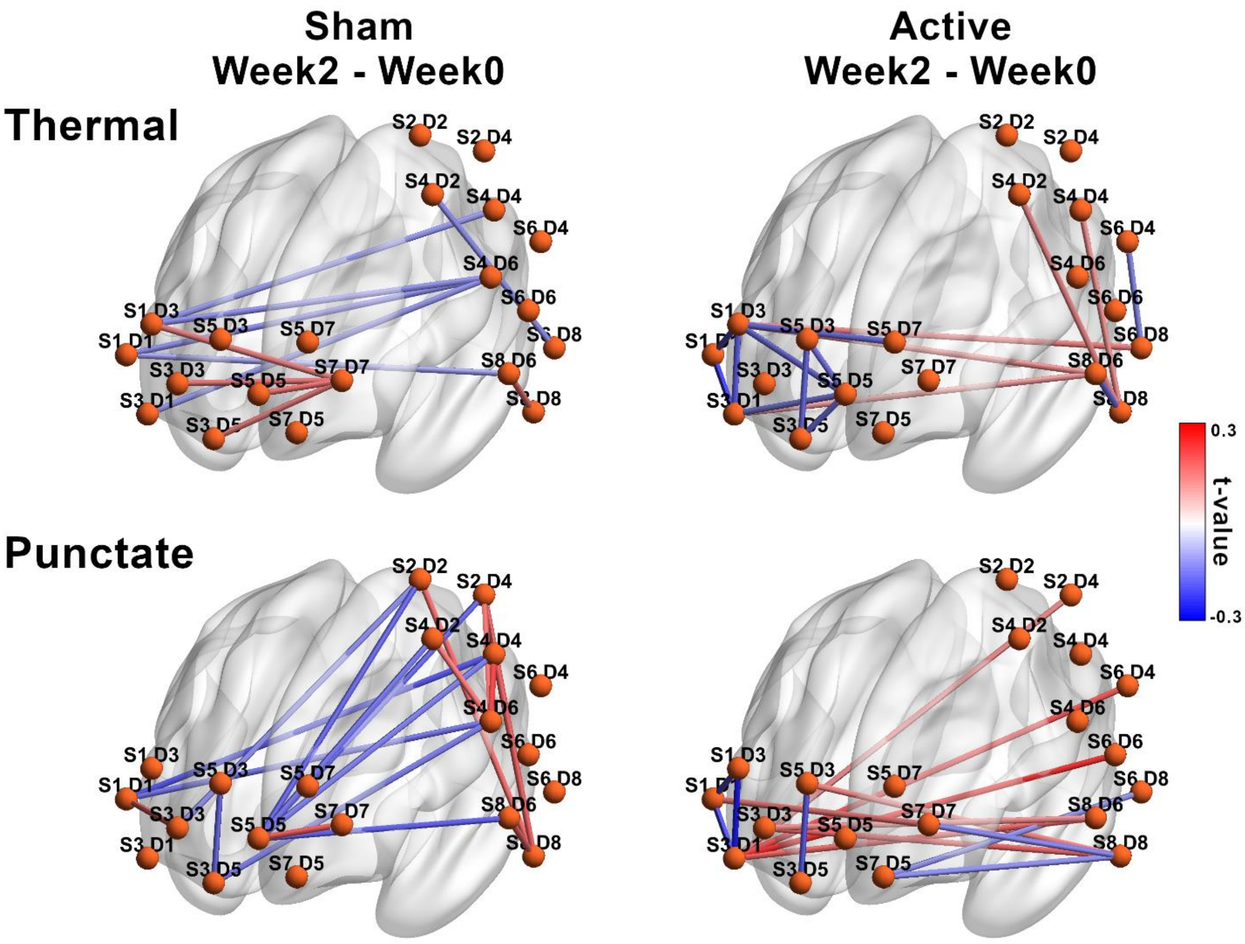

Figure S2 Group-level connectivity maps contrast Week 2 -Week 0 for thermal and punctate stimuli (rows) and sham and active groups (columns). Positive and negative t values are color encoded in the network links.

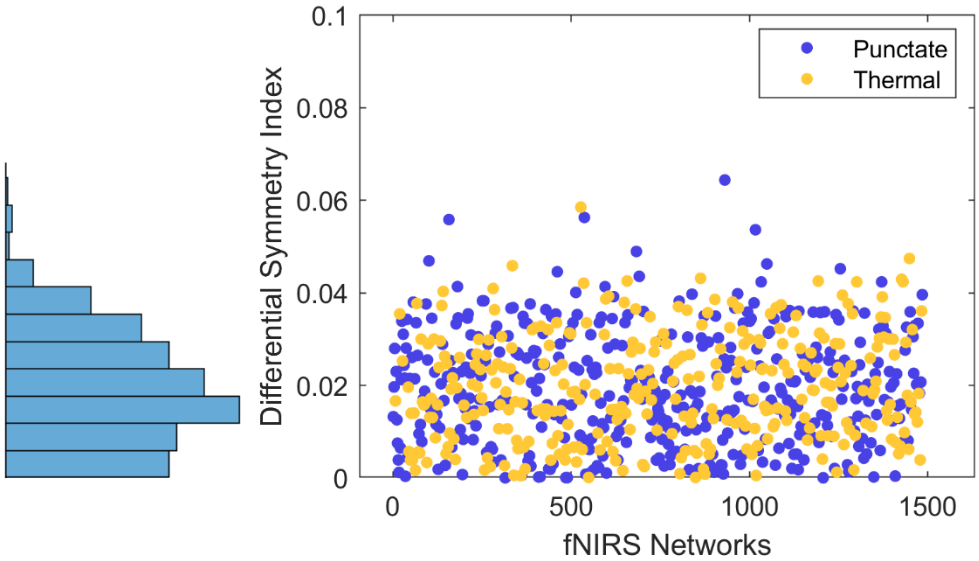

Figure S3 Symmetry between connectivity networks. Differential symmetry index (DSI) quantified the shared information between 743 pairs of HbO2-HbR connectivity networks (circles). The histogram of the symmetry values is indicated on the left.

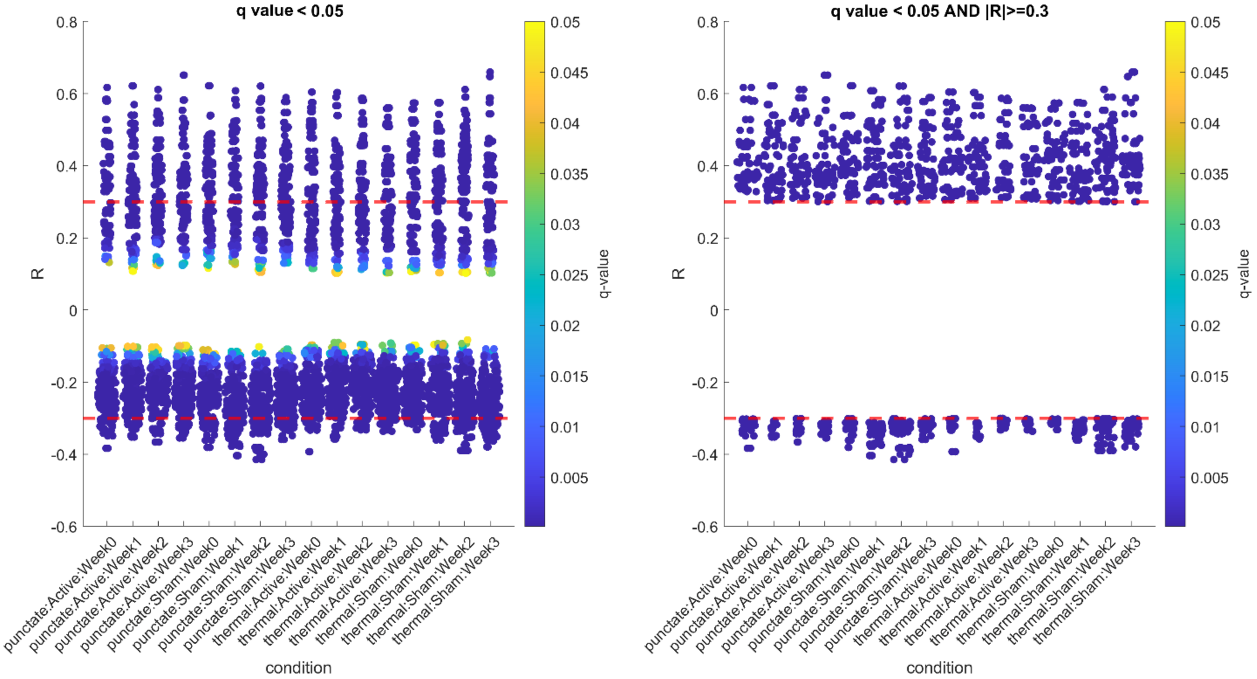

Figure S4 R-values and statistical significance thresholding of functional connectivity networks. R-values (Y-axis) are shown for every functional connection arranged by stimuli, group, and weeks. R-values before (left) and after (right) applying the |R| >=0.3 thresholding. Statistical significance (q-values) of R values is color encoded.

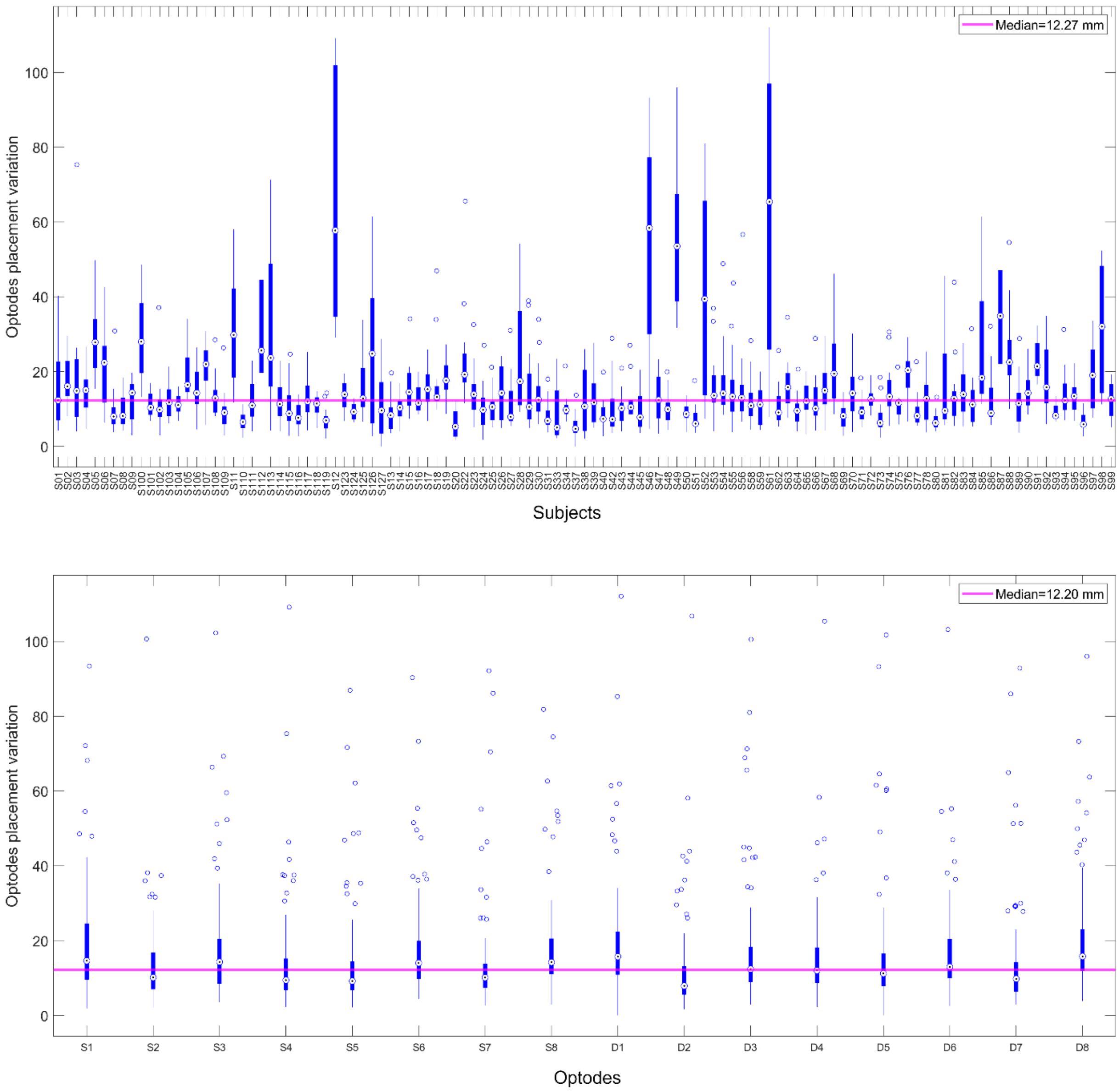

Figure S5 Probe placement variation at the subject level across sessions (top) and at the optode level across optodes (bottom). The outliers and boxes illustrate unsuccessful digitizations with greater IQR.
